# Tumor polyamines as guest cues attract host-functionalized liposomes for targeting and hunting via a bio-orthogonal supramolecular strategy

**DOI:** 10.7150/thno.80857

**Published:** 2023-01-01

**Authors:** Qian Cheng, Zhiqing Yang, Xingping Quan, Yuanfu Ding, Junyan Li, Ziyi Wang, Yonghua Zhao, Xiaoyuan Chen, Ruibing Wang

**Affiliations:** 1 State Key Laboratory of Quality Research in Chinese Medicine, Institute of Chinese Medical Sciences, University of Macau, Taipa, Macau 999078, China.; 2 Departments of Diagnostic Radiology, Surgery, Chemical and Biomolecular Engineering and Biomedical Engineering, Yong Loo Lin School of Medicine and Faculty of Engineering, National University of Singapore, Singapore, 119074, Singapore.

**Keywords:** active target, polyamines, bio-orthogonal, supramolecular homing, host-guest interaction.

## Abstract

Inspired by the attractions of fruit flies to polyamines of rotten food, we developed a facile, bio-orthogonal, supramolecular homing and hunting strategy, relying on the elevated levels of polyamines in tumor as the natural guest cues to attract cucurbit [7] uril (CB[7]) functionalized liposomes to the tumor site, owing to the strong, bio-orthogonal host-guest interactions between CB[7] and polyamines. This supramolecular homing enabled a high targeting efficiency of CB[7] functionalized liposomes, and allowed better tissue penetration and retention in breast tumor. The employment of a receptor functionalized nanomedicine for direct tropism towards endogenous biomarkers as guest cues, reminiscent of natural chemotaxis but in a bio-orthogonal manner, has not been previously reported, offering new sights to the design and development of new nanoformulations that rely on bio-orthogonal interactions for chemotaxis-guided targeting.

## Introduction

Developing targeting strategies holds promise to increase specific drug delivery to cancer cells. Current technologies often rely on either passive or active targeting to deliver the payload to a specific site, thus decreasing the systemic side-effect and improving therapeutic benefits.^1^-^3^ Compared with low-efficiency passive targeting mediated via EPR effects, active biological recognition by antibodies or other biomolecules, mediated via substrate/receptor interactions, to direct drugs to specific sites is often employed for enhancing the targeting efficiency.^3^, ^4^ According to the receptors over-expressed on the surface of tumor cells, specific ligands, such as antibodies, proteins, peptides, aptamers or folic acid, were introduced for the surface modification of liposomes to achieve active targeting via biological substrate/receptor (antibody/antigen) recognition.^5^, ^6^ However, these active ligands remain relatively non-specific, as they could interact with non-target receptors available on other cells or tissues. Furthermore, the “overexpression” of relevant receptors on the surface of tumors is not sufficient to impart high selectivity in general (10^5^-10^6^ versus 10^4^-10^5^ receptors per cell), accompanied by tumor heterogeneity and enormous individual differences from patient to patient, resulting in low cancer targeting efficiency.^7^-^10^ Hence, only several active targeting nanoparticle formulations are in active clinical trials.^11^

Bio-orthogonal chemistry, which refers to a high specificity between specific binding partners (both are often artificial) with relatively few interactions with nontarget biomolecules and little interference with biological processes, provides an ideal approach for improving their tumor-targeting efficiency.^12^, ^13^ For example, metabolic glycoengineering with unnatural sugars provides a powerful tool to label cell membranes with chemical tags for subsequently targeted conjugation of molecular cargos via efficient bio-orthogonal chemistries.^14^ Cucurbit[n]uril family, as synthetic host molecules, have shown great potential in bio-orthogonal chemistry, attributed to the specific high binding affinity between hosts and selected guest molecules, offering a new paradigm for targeted recognition *in vitro* and *in vivo* delivery.^15^-^17^ For instance, Webber and coworkers deposited a cucurbit[7]uril (CB^7^) based hydrogel as a supramolecular pre-targeting cue in the tumor, to enable subsequent homing of a guest-modified chemotherapeutic agent, mediated via bio-orthogonal host-guest interactions.^18^ Kim et al. leveraged an ultrastable synthetic host-guest pair between CB^7^ and adamantyl-(ADA) guests for the specific and bio-orthogonal bioimaging *in vivo*.^19^ Furthermore, Wang et al. constructed an artificial target using CB^7^-capped Fe_3_O_4_ NPs, that were first magnetically deposited into the tumor, subsequently, ferrocene (Fc)-capped Au NPs were specifically delivered to Fe_3_O_4_ NPs, where supramolecular aggregates of these NPs were formed *in situ* mediated via strong, multipoint, CB^7^-Fc host-guest interactions for enhanced CT imaging, and locally activated, tumor-specific photothermal therapy.^20^ Through pre-depositing ADA onto the surface of mitochondria, Wang et al. also demonstrated that CB^7^-grafted hyaluronic acid could recognize, latch and aggregate ADA-modified mitochondria to induce mitochondrial aggregation and fusion intracellularly.^21^ Despite these success, all these prior examples of bio-orthogonal targeting need two steps to complete (pre-depositing an artificial target first, followed by administration of the artificial-ligand (or its modified nanomedicine), which would require a delicate design and laborious preparation of two species for ordered administration. One-step administration for direct bio-orthogonal targeting has been highly desirable.

Polyamines, including spermine (SPM), spermidine (SPD), and putrescine (PUT), which are essential for the growth of tumor cells and can be dysregulated in tumors, are considered as over-expressed tumor biomarkers in several types of cancers including breast and prostate cancers, and their levels increase with the grade of malignancy.^22^-^25^ Thus, polyamines might have potential as therapeutic targets in the prevention and treatment of cancer.^26^ It's worth noting that these polyamines, such as SPM, hold a high binding affinity with CB^7^.^22^ Inspired by the attractions of fruit flies to rotten food via specific recognition of polyamines, herein, instead of pre-targeting a cue in the cancer tissue, polyamines in tumor microenvironment can directly act as an artificial target to attract CB^7^-modified liposomes to the tumor site via bio-orthogonal supramolecular interactions, reminiscent of attraction of fruit flies by polyamines released from rotten food, which may improve the targeting efficiency in the complex biological environment, increase drug accumulation in the tumor, promote tumor penetration and cellular internalization, and ultimately achieve significantly improved therapeutic outcomes and safety profile.

## Results and Discussion

### Preparation and characterization

**Figure 1:**
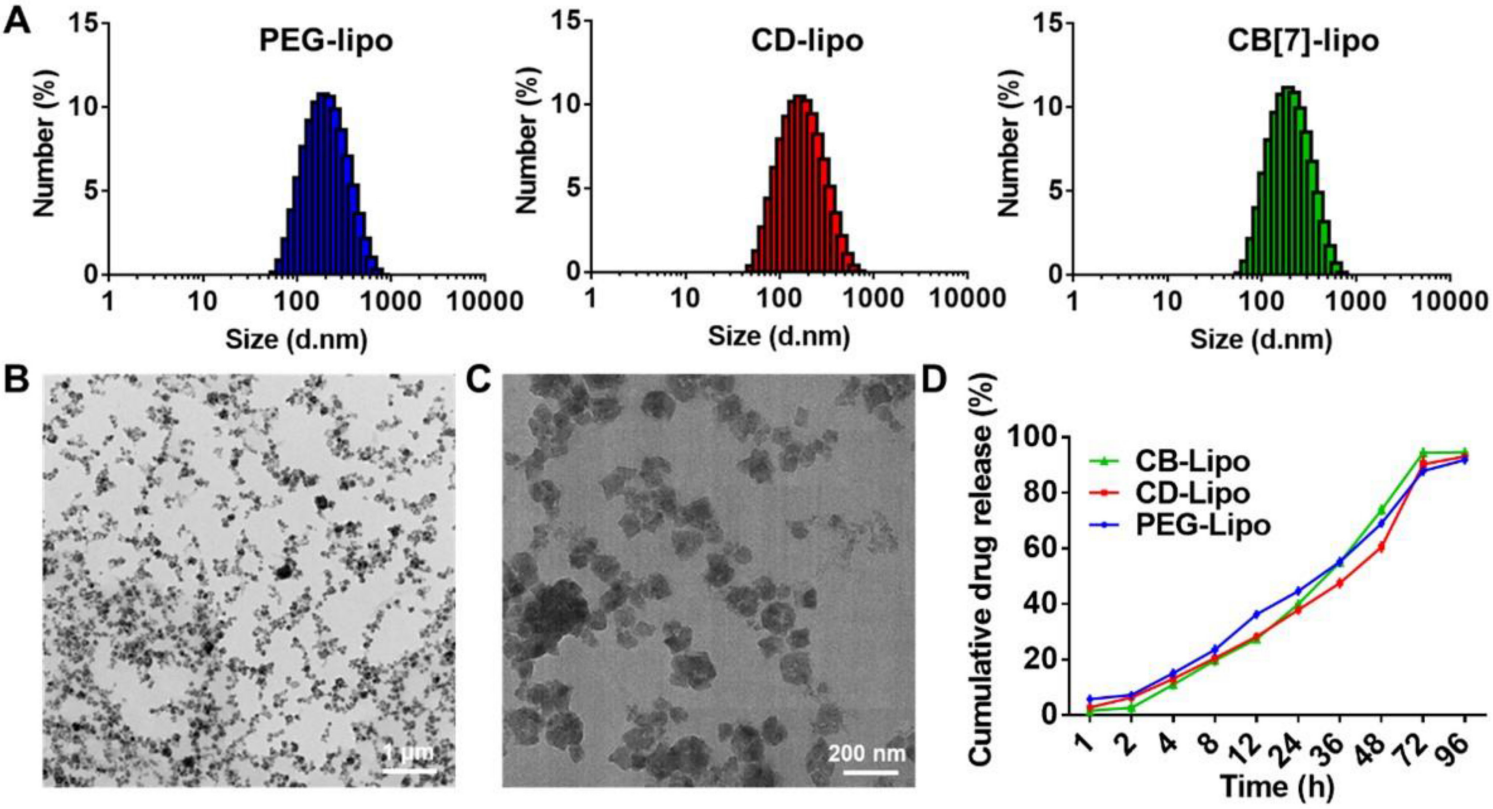
** Preparation and characterization of liposomes. (A)** Size distribution of PEG-lipo, CD-lipo and CB^7^-lipo, respectively, determined by DLS. **(B)** Representative TEM image of CB^7^-lipo. **(C)** Enlarged TEM image of CB^7^-lipo. **(D)** The drug release profiles of DOX loaded PEG-lipo, CD-lipo and CB^7^-lipo, respectively, in PBS medium.

To prepare DSPE-PEG-CB^7^, 1, 2-distearoyl-sn-glycero-3-phosphoethanolamine-Poly (ethylene glycol) (DSPE-PEG) was conjugated to monoallyloxy CB^7^ via thiol-ene click reaction between DSPE-PEG-SH and monoallyloxy CB^7^ and the chemical structure of DSPE-PEG-CB^7^ was shown in Figure S1. As shown in Figure S2, ^1^H NMR spectrum contains the characteristic resonances of CB^7^ at 4.19, 5.35, 5.51 and 5.69 ppm, of PEG at 3.32 ppm, and of DSPE at 1.18 ppm, indicating the successful preparation of DSPE-PEG-CB^7^. Furthermore, the molecular weights (Mw) of the polymers were determined by GPC to be ca. 1753 kDa (DSPE-PEG-SH) and 2534 kDa (DSPE-PEG-CB^7^), respectively, indicating the successful grafting of CB^7^ onto the DSPE-PEG chain. Additionally, in FT-IR spectra of DSPE-PEG-CB^7^, the disappeared peaks at 1600 cm^-1^ from the C=C of monoallyloxy CB^7^ and that at 1475 cm^-1^ corresponding to C-N stretching vibration of CB^7^ was observed, further confirming the successful synthesis of DSPE-PEG-CB^7^ (Figure S3)**.** To assess the host-guest interaction between CB^7^ and polyamines, isothermal titration calorimetry (ITC) was first employed. As shown in Figure S4, CB^7^ exhibited strong binding affinities toward SPM, SPD, and PUT, with association constants (*K*_a_) of (7.95±0.084) ×10^5^ M^-1^, (2.02±0.033) ×10^5^ M^-1^, and (1.45±0.014) ×10^5^ M^-1^, respectively, ensuring the supramolecular tropism of CB^7^ towards these substrates in the complex physiological environment. In addition, ^1^H NMR titration shown in Figure S5 exhibited the binding behavior of the complex. In ^1^H NMR spectra of the CB 7 host-guest complexes in the presence of SPM, the changes of complexation-induced shift can provide valuable information regarding the average position of the guest protons with respect to the CB^7^ cavity. Downfield shifts were observed for deshielded guest protons located adjacent to carbonyl oxygens of the portals (a, b and c), while the shielded guest protons (d and e) located within the CB^7^ cavity exhibited upfield behaviors from those of the free guest, thereby indicating that they are positioned within the cavity of CB^7^. Next, CB^7^ functionalized liposomes were facilely prepared. The CB^7^ modified liposome, CB 7-liposome (CB^7^-lipo), was prepared from soyabean lecithin, cholesterol and DSPE-PEG-CB 7 using a film dispersion method.^27^ To verify the host-guest interaction induced recognition, CD (cyclodextrin)-lipo (constructed by soyabean lecithin, cholesterol and DSPE-PEG-CD), possessing negligible binding affinity with polyamines, and PEG-lipo (constructed by soyabean lecithin, cholesterol and DSPE-PEG), were prepared as controls. Dynamic light scattering and transmission electron microscopy (TEM) analyses demonstrated that these liposomes exhibited similar morphology and a hydrodynamic diameter of ca. 190 nm (Figure 1A-1C). The size distribution of liposomes was monitored over two weeks by DLS (Figure S6), showing no significant change in particle size suggesting a decent stability profile.

For proof of concept, a chemotherapeutic drug, doxorubicin (DOX), was encapsulated into liposomes for potential anticancer applications. The preparation of DOX loaded liposomes was conducted using the same method as described above, by dissolving DOX in deionized water. The drug loading efficiency of DOX loaded PEG-lipo, CD-lipo and CB 7-lipo was 8.1%, 7.8% and 8.5%, respectively, with the corresponding drug encapsulation efficiency of 39.3%, 35.1% and 37.5%, respectively. The drug release kinetics of these liposomes were investigated in PBS at pH 7.4 using HPLC. All the liposomes exhibited undifferentiated sustained release profiles, with approximately 69.1% (PEG-lipo), 66.7% (CD-lipo) and 72.9% (CB^7^-lipo) accumulated drug release observed, respectively, after incubation for 48 h (Figure 1D).

### *In vitro* cytotoxicity and cellular uptake

**Figure 2:**
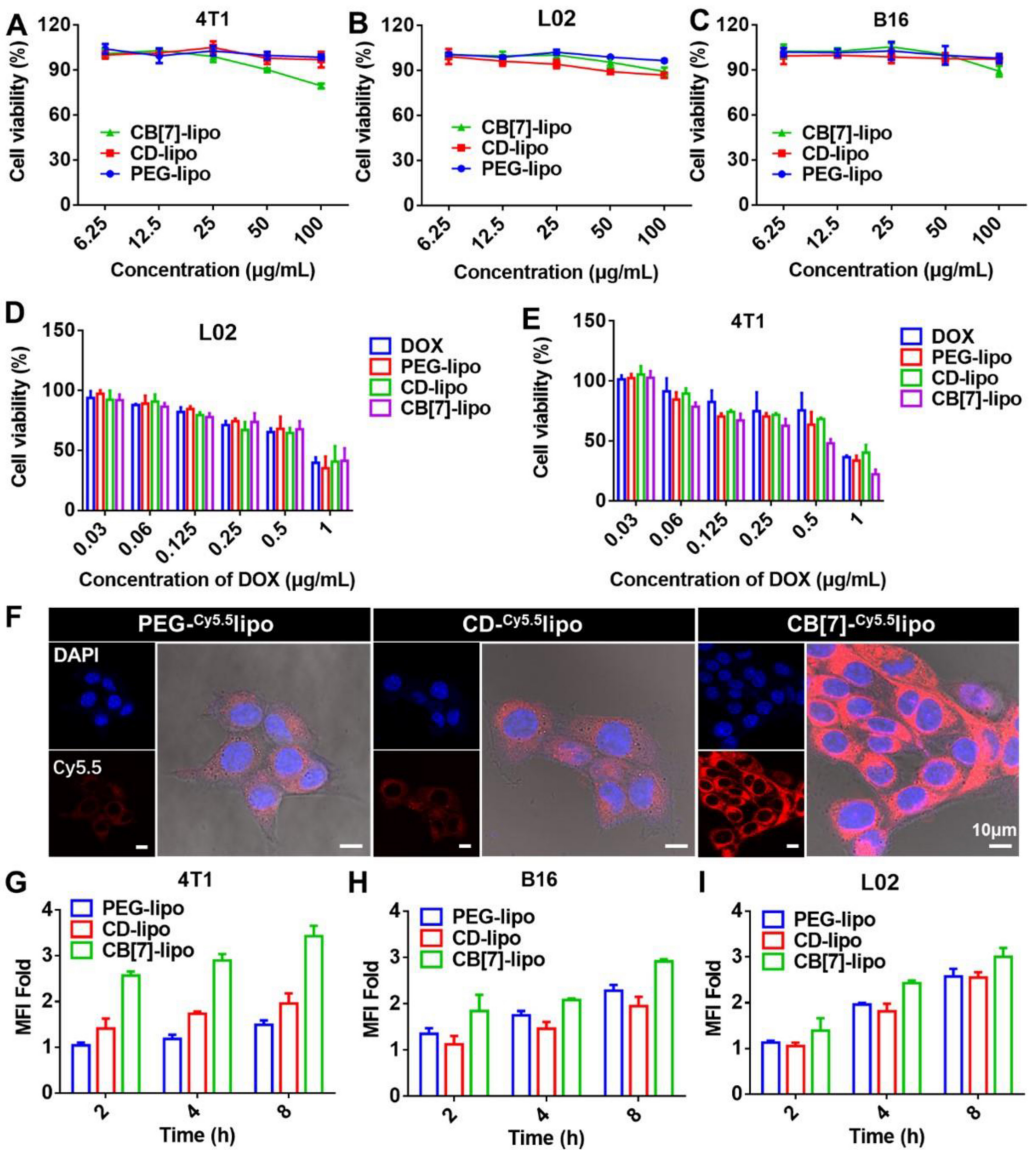
***In vitro* cytotoxicity and cellular uptake behaviors.** Viability of **(A)** 4T1 cells, **(B)** L02 cells and **(C)** B16 cells treated with PEG-lipo, CD-lipo and CB^7^-lipo, respectively, for 48 h. Viability of **(D)** L02 cells and **(E)** 4T1 cells after incubation with free DOX, DOX loaded PEG-lipo, DOX loaded CD-lipo and DOX loaded CB^7^-lipo, respectively, for 48 h. **(F)** CLSM images of 4T1 cells treated with Cy5.5 loaded PEG-lipo, Cy5.5 loaded CD-lipo and Cy5.5 loaded CB^7^-lipo, respectively for 4 h. Mean fluorescence intensities measured by flow cytometry of **(G)** 4T1 cells, **(H)** B16 cells and **(I)** L02 cells after incubation with Cy5.5 labeled PEG-lipo, Cy5.5 labeled CD-lipo and Cy5.5 labeled CB^7^-lipo, respectively for different durations.

The *in vitro* cytotoxicity of drug free liposomes including PEG-lipo, CD-lipo and CB^7^-lipo was first evaluated against 4T1 tumor cells (well-known with polyamine-overexpression) and L02 cells, respectively, using MTT (3-[4,5-dimethylthiazol-2-yl]-2,5 diphenyl tetrazolium bromide) assays.^28^ As shown in Figure 2A and 2B, PEG-lipo and CD-lipo had minimal effects on cell proliferation on both 4T1 cells and L02 cells. Interestingly, CB^7^-lipo exhibited modest toxicity against 4T1 cells but not much against L02 cells, with 79.5% and 89.3% cell viability at a high concentration of 100 μg/mL after incubation for 48 h. The apoptosis rate of 4T1 cells also rose to 31.6% with the concentration of CB^7^-lipo was 100 μg/mL (Figure S7), likely attributed to traps of polyamines by CB^7^, which could dysregulate homeostasis of polyamines leading to the decrease of the cell viability.^22^, ^29^, ^30^ B16 melanoma cell line was employed as a control cancer cell line considering that B16 tumor cells are not polyamine over-expressed tumor cells.^31^ As expected, PEG-lipo, CD-lipo and CB^7^-lipo all exhibited negligible antitumor activity within the concentration of 100 μg/mL after incubation for 48 h (Figure 2C). However, all of these liposomes exhibited negligible cytotoxicity against B16 cells, especially when the concentrations were in the low range. Subsequently, the chemotherapeutic drug, DOX, was encapsulated into these liposomes. As shown in Figure 2D and 2E, DOX-loaded CB^7^-lipo showed much higher cytotoxicity than free DOX and DOX-loaded PEG-lipo and CD-lipo against 4T1 cells, whereas these different formulations did not exhibit any difference in cytotoxicity against L02 cells. The corresponding IC_50_ values of the four formulations against the two cell lines were calculated and listed in Figure S8.

Subsequently, we studied the cellular uptake behaviors of liposomes qualitatively and quantitatively by confocal laser scanning microscopy (CLSM) and flowcytometry. In order to better track the fluorescence of liposomes in cell, Cy5.5 dye was loaded into liposomes as a fluorescent tracker. As shown by CLSM images in Figure 2F, CB^7^-lipo exhibited a faster cellular uptake with bright red fluorescence in the 4T1 cells observed after incubation for 4 h. In contrast, PEG-lipo and CD-lipo treated 4T1 cells had significantly weaker red fluorescence. Via flow cytometry analysis (Figure 2G), the mean fluorescence intensity in the 4T1 cells incubated with CB^7^-lipo was 2.31-fold and 1.77-fold that of the cells treated with PEG-lipo and CD-lipo, respectively, confirming that CB^7^-lipo possessed faster cellular uptake behaviors by 4T1 cells, likely owing to the supramolecular recognition of intracellular polyamines by CB^7^. Conversely, the cellular uptake of L02 and B16 cells incubated with those three liposomes didn't vary much (Figure 2H and 2I), suggesting that intracellular polyamines of 4T1 resulted in the enhanced uptake of CB^7^-lipo. To verify the tropism effect of CB^7^ to polyamine, to see if the cell binding and internalization of the liposomes could be enhanced by addition of exogenous polyamines, we pre-incubated cells with SPM before adding CB^7^-lipo. Polyamine-overexpressed 4T1 cells were employed to facilitate update of exogenous SPM, as the polyamine transport systems are upregulated in these tumor cells. As shown in Figure S9, after pre-incubation of cells with SPM for 2 h, more cellular uptake of CB^7^-lipo labeled with Cy5.5 was observed, when compared with cells directly incubated with CB^7^-lipo alone. In contrast, no significant increase in cellular uptake of CB^7^-lipo was observed in L02 and B16 cells, because polyamines transportation system is usually highly regulated.^26^

### Transcellular transfer, penetration and supramolecular tropism of CB^7^-lipo

**Figure 3:**
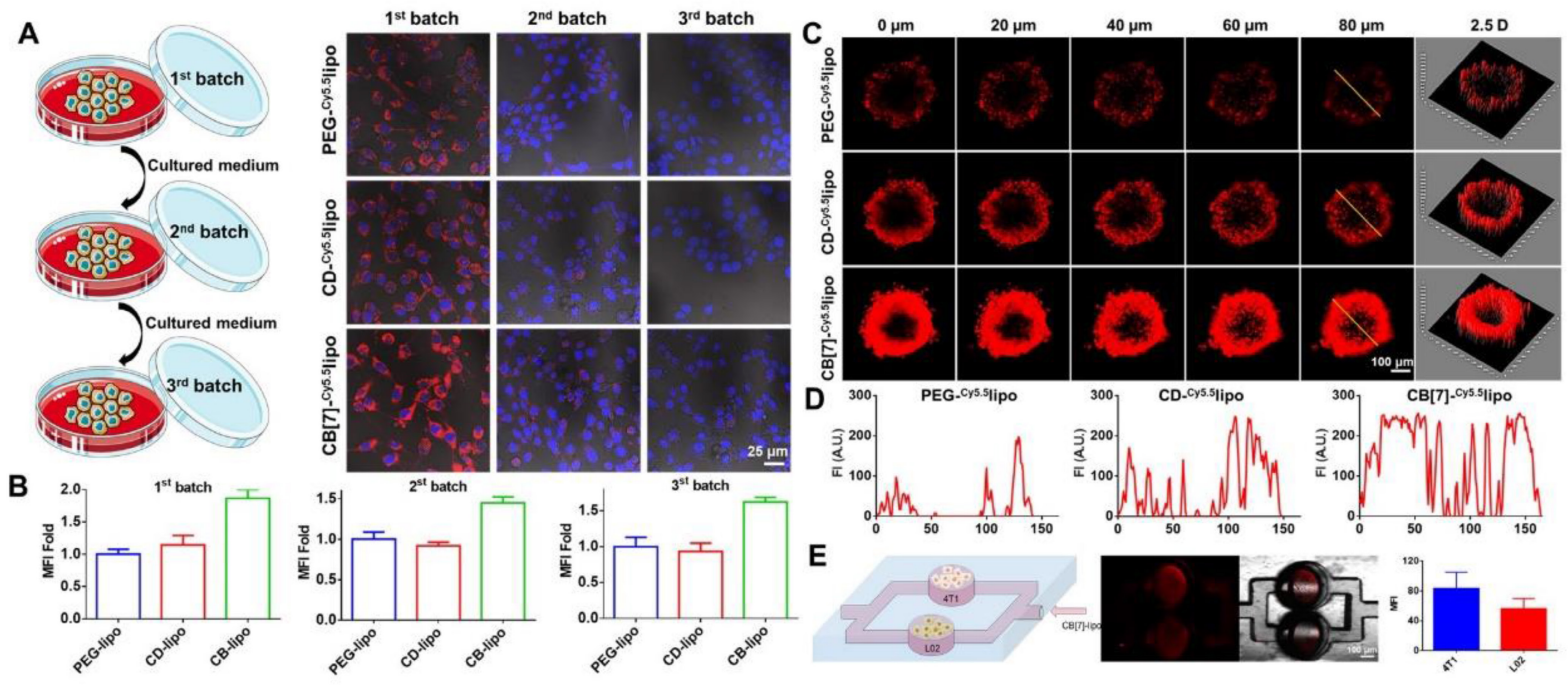
** In *vitro* transcellular transfer, tissue penetration and supramolecular tropism driven targeting to polyamine over-expressed 4T1 cells. (A)** Intercellular transportation of Cy5.5-labeled PEG-lipo, CD-lipo and CB^7^-lipo through 4T1 cells. 4T1 cells (the first batch) were cultured with Cy5.5-labeled PEG-lipo, CD-lipo and CB^7^-lipo, respectively, for 4 h, washed with PBS, and imaged via CLSM; the cells were subsequently cultured in fresh medium for 12 h, and the medium was then harvested to incubate with the second batch of 4T1 cells for 12 h, followed by washing and imaging via CLSM; the same procedures were conducted again to obtain the third batch of cells. **(B)** Quantitative red fluorescence from A. **(C)** 4T1 constructed MTSs were cultured with Cy5.5-labeled PEG-lipo, CD-lipo and CB^7^-lipo, respectively, for 12 h. The MTSs were visualized using CLSM in Z-stacks with 20 µm intervals. **(D)** The Cy5.5 fluorescence intensity along with the randomly selected yellow line across different liposomes treated MTSs. **(E)** Scheme and CLSM images of microchip assay. 4T1 cells and L02 cells were seeded in the two separate channels of a microchip, respectively. Cy5.5 labeled CB^7^-lipo was infused from the vertical channel and allowed to flow for half an hour before CLSM analysis and quantitative analysis.

Next, we examined if the accelerated internalization of CB^7^-lipo would induce higher transcellular transportation that will benefit tumor penetration. 4T1 cells were treated with PEG-lipo, CD-lipo and CB^7^-lipo for 4 h, respectively. The liposomes outside cells were washed off, and the cells were incubated for another 12 h with fresh medium. Thereafter, the incubation medium was collected and incubated with next batch of fresh cells. These procedures were performed for two rounds in sequence. As shown in Figure 3A and 3B, red fluorescence could be observed in all three batches of cells treated with Cy5.5 loaded CB^7^-lipo. In contrast, the signals from PEG-lipo and CD-lipo were significantly attenuated in the third batch of cells, indicating the transcellular transportation of CB^7^-lipo was faster than those liposomes without CB^7^. The promoted transcellular transfer occurred as the joint result of CB^7^ enhanced cellular internalization and concomitant exocytosis, which was associated with the strong supramolecular tropism of CB^7^ to intracellular polyamines.

Subsequently, we further studied the penetration capability of liposomes using 4T1 cells constructed multicellular tumor spheroids (MTSs). When the 4T1 MTSs grew to a diameter of ~ 300 nm, the MTSs were incubated with Cy5.5 labeled PEG-lipo, CD-lipo and CB^7^-lipo, respectively, for 12 h, and analyzed via CLSM. As shown in Figure 3C and 3D, weak fluorescence could be observed in the periphery but not the interior of MTSs treated with PEG-lipo group and CD-lipo. In contrast, CB^7^-lipo treated MTSs exhibited much brighter Cy5.5 fluorescence penetrated from the periphery deep into the core, suggesting that CB^7^ facilitated the penetration of liposomes into tumor tissues (MSTs).

Encouraged by the excellent transcellular transportation and tissue penetration capability with 4T1 tumor cell models, we further verified the supramolecular tropism of CB^7^-lipo using a microfluidic chip. 4T1 and L02 cells were seeded in the two separate channels of the microchip, respectively (Figure 3E left). Cy5.5 labeled CB^7^-lipo was prepared in the vertical channel and allowed to flow for half an hour. As shown by CLSM and quantitative analysis (Figure 3E), significant difference in fluorescence intensity of the two channels was observed. CB^7^-lipo accumulated more in the channel seeded with 4T1 cells, than that seeded with L02 cells, indicating the targeted accumulation of CB^7^ modified liposomes to the polyamine overexpressed cancer cells. To confirm that it was polyamines that induced the targeting effect, we measured the intracellular concentration of polyamine in 4T1 and L02 cells, respectively, via HPLC. As shown in Figure S10, the intracellular concentrations of SPM, SDP and PUT in 4T1 cells were 4.3-fold, 2.4-fold, 6.6-fold higher than those in L02 cells, respectively, consistent with the previously reported results.^29^ This result quantitatively explains why CB^7^-lipo has a stronger targeting ability to 4T1 cells. The host-guest recognitions may drive the targeting of CB^7^-lipo to 4T1 cells/tissues, thereby promoting the cellular uptake, transcellular transfer and penetration.

### *In vivo* biodistribution

**Figure 4:**
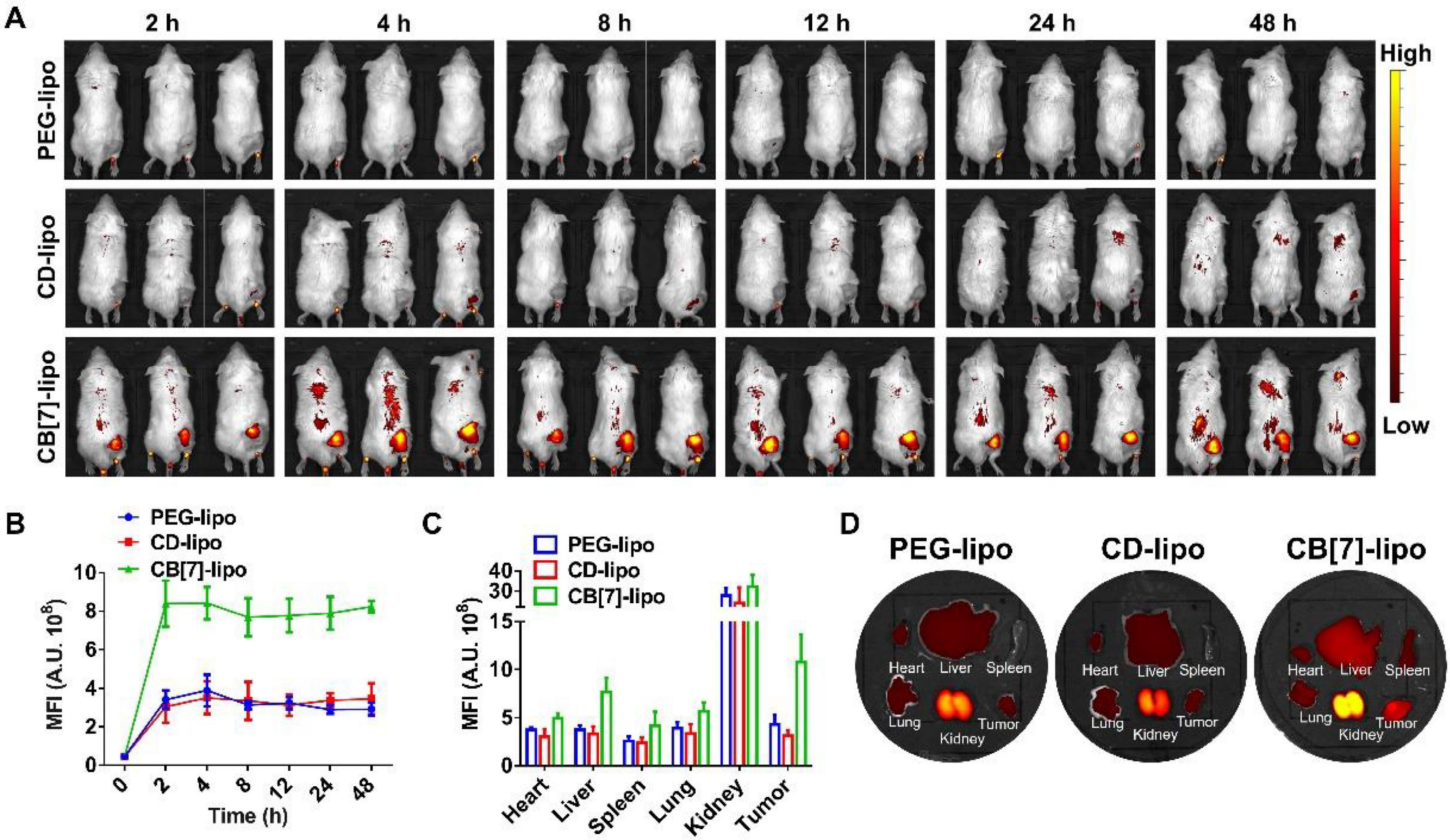
** Biodistribution of Cy5.5 labeled CB^7^-lipo, PEG-lipo and CD-lipo, respectively, in 4T1 tumor-bearing mice. (A)** *In vivo* real-time imaging of tumor-bearing mice after intravenous injection of Cy5.5 labeled PEG-lipo, CD-lipo and CB^7^-lipo (Cy5.5-eq. dose of 0.5 mg kg^-1^). **(B)** The mean Cy5.5 fluorescence intensities of the tumors at different time points after intravenous injection of Cy5.5 labeled PEG-lipo, CD-lipo and CB^7^-lipo, respectively. **(C)** Quantification of the mean Cy5.5 fluorescence intensity in the organs and tumors. **(D)** Ex vivo IVIS images of the heart, liver, spleen, kidneys, lungs, and tumor harvested from the mice after 48 h post injection.

To evaluate the targeting effects of CB^7^-lipo in the tumor tissue *in vivo*, Cy5.5 loaded CB^7^-lipo and PEG-lipo and CD-lipo were intravenously injected into 4T1 tumor-bearing balb/c mice and monitored by an IVIS system at scheduled time points. As shown in Figure 4A, CB^7^-lipo exhibited a much higher accumulation in the tumor site of mice, when compared with that of PEG-lipo and CD-lipo treated mice at 2 h post-injection, and the signal intensity of CB^7^-lipo in tumor site reached maxima, in a much faster manner than PEG-lipo and CD-lipo. As shown in Figure 4B, at each test time point, the signal intensity of CB^7^-lipo in the tumor site was higher than that of PEG-lipo and CD-lipo. The fluorescence intensity of CB^7^-lipo in the tumor was approximately 2.5-fold and 3.4-fold that of PEG-lipo and CD-lipo, respectively, at 48 h post-injection (Figure 4C). After 48 h post-treatment, the mice were sacrificed, and the major organs and tumor tissues were excised for ex vivo imaging (Figure 4D), the tumor of CB^7^-lipo treated mice exhibited much brighter fluorescence than those of PEG-lipo and CD-lipo treated groups. These results demonstrated that CB^7^-lipo could accumulate more efficiently in the tumors, likely owing to the supramolecular tropism of CB^7^ on the liposomes towards polyamines in the 4T1 tumor.

To explore the mechanism that facilitates the increased cell surface adsorption as well as increased accumulation of CB^7^-lipo in various tissues and organs, the zeta potential of PEG-lipo, CD-lipo and CB^7^-lipo with and without the presence of SPM were measured. As shown in Figure S11, PEG-lipo, CD-lipo and CB^7^-lipo all presented negative surface charges of -27.02 mV, -18.03 mV and -16.03 mV, respectively. As polyamines mainly exist intracellularly^26^, a small amount (micromolar) of polyamine was used to mimic polyamine concentrations *in vitro*. After adding SPM, the PEG-lipo and CD-lipo presented similar surface charges of -27.31 mV and -17.37 mV, respectively, remaining largely unchanged. However, an obviously reduced negative surface charge was obtained in CB^7^-lipo valued at -7.1 mV after binding with SPM. Therefore, it is possible that the reduced negative surface charges offered by encapsulating polyamines facilitate the increased cell surface adsorption as well as accumulation in different tissues, organs, and tumor site.

### Antitumor efficacy

**Figure 5:**
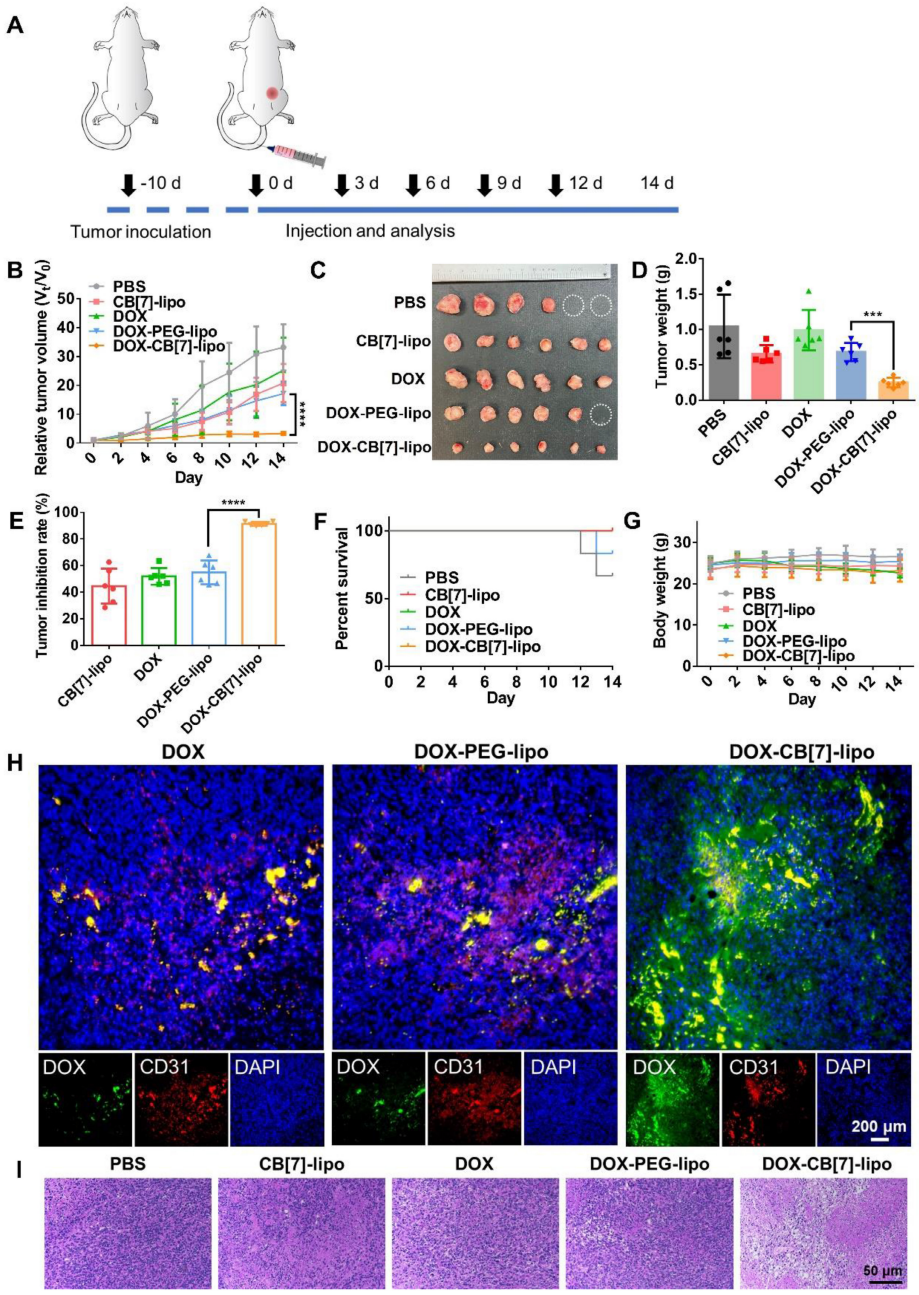
** The antitumor efficacy of CB[7]-lipo against 4T1 tumor bearing balb/c mice. (A)** Scheme showing the treatment protocol in mice. 4T1 tumor bearing balb/c mice were divided into 5 groups intravenously administered with different treatment (n = 6 for each group) of: PBS, blank CB[7]-lipo, free DOX (DOX), DOX loaded PEG-lipo (DOX-PEG-lipo) and DOX loaded CB[7]-lipo (DOX-CB[7]-lipo) at DOX equivalent dose of 4 mg/kg every 3 days for a total of five treatments. **(B)** Tumor growth curves of the mice. **(C)** Photographs of the tumors resected at the end of the experiment. Tumors exceeding the ethically limited size were circled in white. **(D)** Tumor weight. **(E)** Tumor inhibition rate. **(F)** Survival rate. and **(G)** Body weight changes during the 14-d treatment. **(H)** The immunofluorescence staining images showing the intratumoral distribution of DOX, from tumors harvested by the end of 14 d observation. The tumor blood vessels were stained with CD31 and APC-labeled secondary antibodies (red), and cellular nuclei were stained with DAPI (blue). **(I)** H&E staining of the tumor tissues harvested by the end of 14 d observation.

The antitumor activities of these liposomes were evaluated in a 4T1 tumor model. Tumor-bearing balb/c mice were randomly divided into five groups and intravenously administered with PBS, blank CB^7^-lipo (CB^7^-lipo), free DOX (DOX), DOX loaded PEG-lipo (DOX-PEG-lipo) and DOX loaded CB^7^-lipo (DOX-CB^7^-lipo) at equivalent DOX dose of 4 mg/kg once every 3 days for a total of five treatments (Figure 5A). The tumor volume and body weight were measured every other day. As shown in Figure 5B-5D, among the five groups of mice, the tumor size of PBS treated mice grew fastest to nearly approximately 33-fold that of the original size with the heaviest tumor weight. Each DOX formulation showed varying degrees of tumor suppression with slower growth of tumor volumes when compared with the PBS treated mice. Remarkably, blank CB^7^-lipo showed an obvious tumor inhibitory effect, with a tumor inhibition rate of 44.6%, likely attributed to the supramolecular trap of polyamines through inclusion in the host cavity,^29^ in line with the previously described *in vitro* data. Benefitting from the synergistic effect of enhanced targeting and interference with polyamine activity, DOX-loaded CB^7^-lipo exhibited the highest tumor suppression effect (91.4%) than the DOX (49.9%) and DOX-loaded PEG-lipo (56.8%) (Figure 5E). Furthermore, the mice treated with CB^7^-lipo, DOX and DOX-CB^7^-lipo all survived the 14 days experiment, owing to the limited tumor development, in a contrast to 66.7% and 83.3% survival rates of the mice from PBS and DOX-PEG-lipo treated groups, respectively (Figure 5F). Additionally, the mice treated with free DOX showed moderate weight loss, suggesting the non-specific toxicity of the chemotherapeutic agent. Conversely, other four formulations didn't cause significant fluctuation in their body weight during the 2-week follow up (Figure 5G), indicating that all formulations were well-tolerated. To examine the retention of DOX in the tumor site, at the end of treatment, the tumor tissue was collected and stained for immunofluorescent analysis. The tumor blood vessels were stained with CD31 and APC-labeled secondary antibodies (red), and cellular nuclei were stained with DAPI (blue). As shown in Figure 5H, the green fluorescence from DOX and DOX-PEG-lipo treated groups was mainly restricted to the blood vessels, indicating inefficient diffusion into the deep tumor tissue. Instead, the green fluorescence from DOX-CB^7^-lipo treated mice was well perfused in the tumor tissue with even distribution, suggesting that DOX-CB^7^-lipo was able to efficiently penetrate into the deep tumor space. These findings suggest a mechanism that the targeting and retention was afforded by high-affinity host-guest recognition. Furthermore, the histological analysis of the tumor tissue sections showed abundant apoptotic cells with shrinking and nuclear damage from DOX- CB^7^-lipo treated group, while negligible cancer cell damage was observed in the other four groups (Figure 5I). In addition, all H&E of the major organs showed good biocompatibility in all five formulations (Figures S12), indicating the decent biosafety profiles of these formulations. We also examined the effects of these formulations on the hematological parameters and liver/kidney function biomarkers including ALT (alanine transaminase), AST (aspartate transaminase), BUN (blood urea nitrogen), CREA (creatinine), UA (uric acid), WBC (white blood cells), RBC (red blood cells), HGB (hemoglobin), HCT (hematocrit), MCV (mean corpuscular volume), MCH (mean corpuscular hemoglobin), MCHC (mean corpuscular hemoglobin concentration), RDW (red blood cell volume distribution width), PLT (platelets) and MPV (mean platelet volume). As shown in Figure S13, little changes were observed when compared with the control group.

## Conclusion

In summary, we simply utilized tumor over-expressed polyamines in the tumor microenvironment as natural guest cue, for the first time, to attract CB^7^-lipo to the tumor site owing to the strong bio-orthogonal host-guest interactions between CB^7^ and polyamines. Benefitting from the supramolecular homing effects, CB^7^-lipo exhibited excellent cancer targeting and rapid cancer cell internalization, and allowed better tissue penetration and retention of such liposomes in the polyamine-overexpressed tumor both *in vitro* and *in vivo*. Reminiscent of natural chemotaxis, this work provides a facile supramolecular bio-orthogonal strategy for targeted drug delivery, offering new solutions to potentially treat some refractory malignant tumors with rare targets such as triple-negative breast cancer, brain glioma and so on, and also providing new sights to the design and development of new nanoformulations that rely on bio-orthogonal interactions for targeted delivery.

## Supplementary Material

Supplementary methods and figures.

## Figures and Tables

**Scheme 1:**
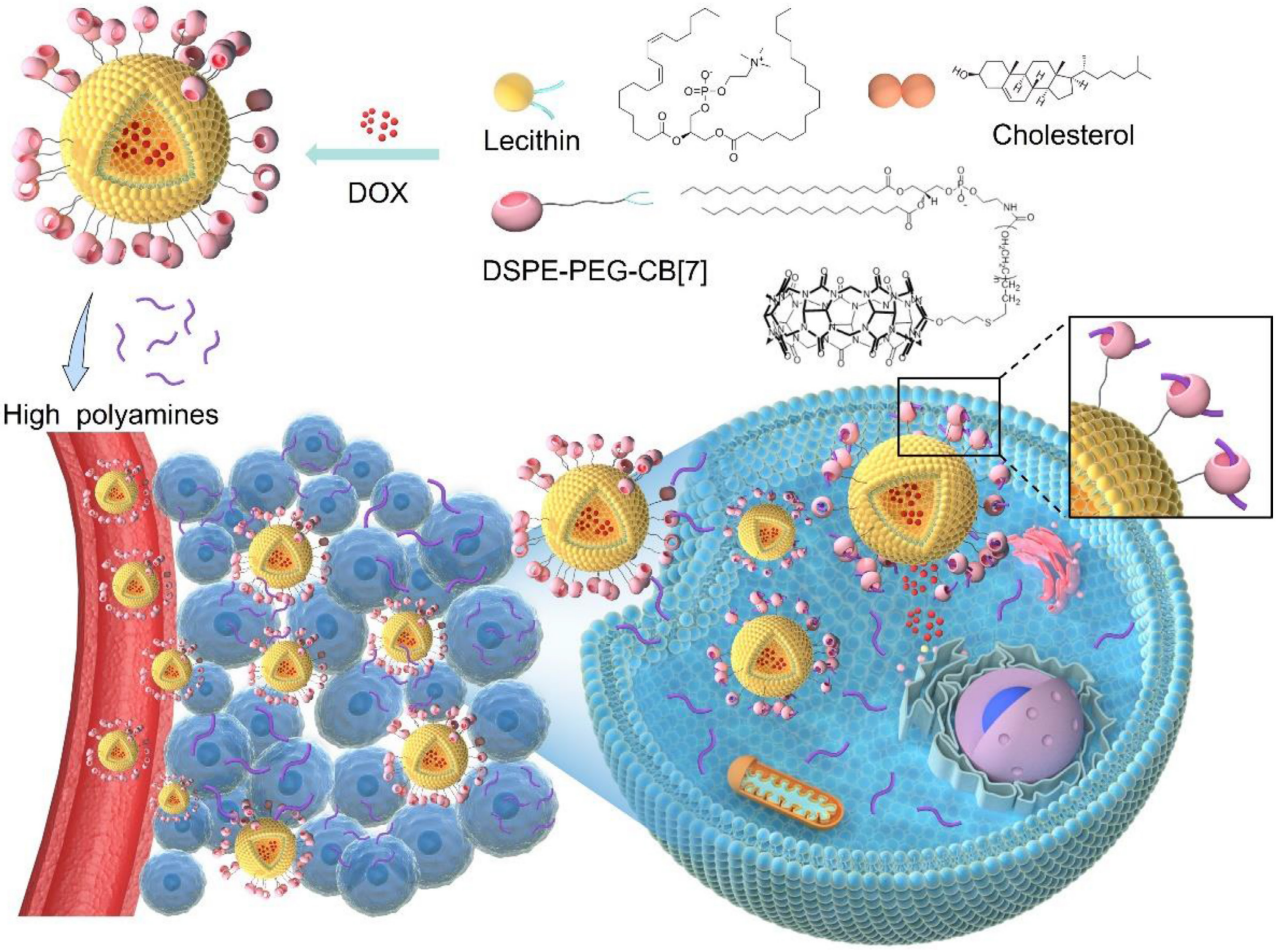
Utilizing polyamines enriched in the tumor microenvironment as a guest cue to attract CB^7^-modified liposomes for enhanced targeting and hunting via bio-orthogonal supramolecular strategy, reminiscent of attraction of fruit flies by polyamines released from rotten food.
