# Supplementary material for: Tumor polyamines as guest cues attract host-functionalized liposomes for targeting and hunting via a bio-orthogonal supramolecular strategy
Source: Theranostics. 2023 Jan 1;13(2):611–20. doi: 10.7150/thno.80857 (PMC9830427; doi:10.7150/thno.80857)
Supplement: Supplementary file 1 — Supplementary methods and figures. [file thnov13p0611s1.pdf]

Tumor polyamines as guest cues attract host-functionalized liposomes for targeting and hunting via a bio-orthogonal supramolecular strategy

Qian Cheng1[a], Zhiqing Yang1[a], Xingping Quan[a], Yuanfu Ding[a], Junyan Li[a], Ziyi Wang[a], Yonghua Zhao[a], Xiaoyuan Chen*[b], Ruibing Wang*[a]

Abstract: Inspired by the attractions of fruit flies to polyamines of rotten food, we developed a facile, bio-orthogonal, supramolecular homing and hunting strategy, relying on the elevated levels of polyamines in tumor as the natural guest cues to attract cucurbit[7]uril (CB[7]) functionalized liposomes to the tumor site, owing to the strong, bio-orthogonal host-guest interactions between CB[7] and polyamines. This supramolecular homing enabled a high targeting efficiency of CB[7] functionalized liposomes, and allowed better tissue penetration and retention in breast tumor. The employment of a receptor functionalized nanomedicine for direct tropism towards endogenous biomarkers as guest cues, reminiscent of natural chemotaxis but in a bio-orthogonal manner, has not been previously reported, offering new sights to the design and development of new nanoformulations that rely on bio-orthogonal interactions for chemotaxis-guided targeting.

Experimental Procedures

Materials
Cyanine5.5 (Cy5.5) was purchased from Abcam (China). DSPE-PEG-CB[7] was synthesized in our laboratory according to previous report[1]. 4', 6-Diamidino-2-phenylindole (DAPI) was purchased from Invitrogen (USA). All other chemical reagents were purchased from Aladdin (China). All reagents and solvents were used as supplied without further purification.

The isothermal titration calorimetry test was carried out on a Malvern MicroCal PEAQ-ITC. Cellular uptake was analyzed by a FACS flow cytometer (Beckman coulter). A confocal laser scanning microscopy (CLSM, Zeiss LSM710) was used to directly visualize the intracellular location of liposomes. Cellular viability was evaluated via MTT assays with the assistance of a multi-mode microplate reader (FlexStation 3). In vivo imaging was performed on in vivo imaging system (Lumina XR III). NMR signals were recorded on a Bruker NMR instrument (Ultra Shield Plus 400 MHz). Gel permeation chromatography (GPC) analysis was performed on waters1525 & Agilent PL-GPC220. Fourier transform infrared spectroscopy (FT-IR) was performed on an IR spectrometer (Spectrum Two, PerkinElmer) using potassium bromide in the scanning wavenumber range of 4000 to 1000 cm^{-1} for the diffuse reflectance spectra.

B16 cells, L02 cells, and 4T1 cells were obtained from American Type Culture Collection (ATCC; USA). All of these cell lines were authenticated by DNA fingerprinting, isozyme detection, viability test, and mycoplasma detection. Male C57BL/6 mice (6 weeks) were purchased from Faculty of Health Sciences, University of Macau. All animal procedures were approved by the Animal Ethics Committee, University of Macau and were conducted in accordance with the Animal Management Rules of the Ministry of Health of the P. R. China.

Preparation of liposomes
liposome was prepared by a film hydration method.[2] Briefly, lecithin (36 mg), cholesterol (16 mg), and DSPE-PEG/DSPE-PEG-CD/DSPE-PEG-CB[7] (14 mg) (mole ratio 47:41:4) were dissolved in 4 mL of dichloromethane. The organic solvent was subsequently evaporated under reduced pressure by using a rotary evaporator to form a thin transparent film on the wall of the flask. After being dried under vacuum to eliminate the residual solvent, the film was dissolved with 5 mL of deionized water and then evaporated without pressure for 1.5 h at 55 ℃. Finally, the suspension was manually extruded through filter membrane (200 nm) to form uniform sized PEG-lipo, CD-lipo and CB[7]-lipo. The obtained liposomes were collected and characterized by TEM and DLS. To prepare the DOX-loaded liposomes, DOX in deionized water was added to dissolved film solution, and the following process was same to the liposome preparation. Finally, purified DOX loaded liposomes were obtained via dialysis in deionized water.

DOX release from DOX-loaded liposomes
In vitro DOX release from PEG-lipo, CD-lipo and CB[7]-lipo were analyzed by a dialysis method. 2 mL of PEG-lipo/CD-lipo/CB[7]-lipo solutions with 200 μg/mL of DOX were applied to a dialysis bag (MWCO 1000). The dialysis bag was then immersed in 50 mL of PBS buffer (pH 7.4) in the dark. 0.5 mL of the PBS medium was sampled at timed intervals and re-added an equal amount of fresh medium. The DOX concentration in the medium was determined by HPLC, and the accumulative release amount was calculated.

Cellular uptake behavior
4T1 cells, B16 cells and L02 cells were cultured in confocal dishes in DMEM containing 10% (v/v) FBS at 37°C and then individually incubated in DMEM with Cy5.5 loaded lipo, Cy5.5 loaded CD-lipo and Cy5.5 loaded CB-lipo at a concentration of 0.08 mg/ml DSPE-PEG/DSPE-PEG-CD/DSPE-PEG-CB[7] for different time points (2, 4, and 8 hours) Then, the cells were fixed by paraformaldehyde, and the fluorescence photography was imaged by CLSM. Quantitative analysis was conducted by using FACS flow cytometer.

Cell viability
The cell viability of blank PEG-lipo, CD-lipo and CB[7]-lipo against 4T1, B16 and L02 cell lines were evaluated by MTT assays. 4T1, B16 and L02 cells were seeded into 96-well plates at a density of 5000 cells/well in 100 μL of DMEM containing 10% (v/v) FBS and cultured for 24 h at 37 °C. Blank PEG-lipo, CD-lipo and CB[7]-lipo were dissolved in PBS and diluted to the required concentration and further incubated for 48 h. Then the medium was replaced with DMEM containing MTT and the

survival number of cells was determined by MTT enzyme-linked immunometric meter. The in vitro cytotoxicity of DOX-loaded PEG-lipo, CD-lipo and CB[7]-lipo was evaluated using the same method.

Analysis of intracellular polyamines.
4T1 and L02 cells were seeded into 6-well plates at 1×10^5 cells/well and cultured for 24 h under 5% CO_2 at 37 °C. the cells were harvested by trypsinization, and then were resuspended in PBS. A part of cell suspension was used to determine the protein content according to the BCA assay, and another part of the suspension was added with perchlorate solution to extract the cells. The cell extractive was mixed with hexanediamine and dansyl chloride, and pH was adjusted to 9.5 with saturated sodium carbonate solution, reacting at 50 °C for 30 min. After cooling, ethyl acetate was added to extract the sample solution, and the concentrations of the natural polyamines (SPM, SPD and PUT) were determined by HPLC.

Intercellular transport of liposomes
4T1 cells were seeded in glass-bottom confocal dishes at a density of 1×10^4 cells per dish and incubated for 12 h. The cells (1st batch) were cultured with Cy5.5 loaded PEG-lipo, CD-lipo and CB[7]-lipo for 4 h, washed with PBS, and imaged with CLSM. The cells were then cultured in 1 mL with fresh medium for 12 h, and the medium was harvested to incubate the 2nd batch of cells for 12 h, followed by washing with PBS and imaging with CLSM. The same procedures were implemented for another two rounds.

Penetration of Cy5.5 loaded liposomes in multicellular tumor spheroids
4T1 monolayer cells were plated on MS-9096UZ PrimeSurface3D plates with blank DMEM. About 3 days later, the multicellular tumor spheroid models (MCTS) had formed with a homogeneous diameter around 300 μm. These MCTS were incubated with Cy5.5 loaded PEG-lipo, CD-lipo and CB[7]-lipo, respectively for 12 hours. The images of MCTS were acquired on Inverted Microscope Solution DMi8 S Platform, and the 3D version images were obtained by using the Z-Stack scanning from top to bottom of tumor spheroid at 1 μm interval.

Microchip assay
SPM overexpressed 4T1 tumor cells and SPM normal-expressed L02 cells were seeded in the two branching channels on the chip. The cells were allowed to subside and adherence to the chip for 4 h. Then the Cy5.5 labelled CB[7]-lipo was prepared in the vertical channel and allowed to flow for half an hour, recorded by CLSM.

In vivo targeting efficiency of liposomes in 4T1 tumor bearing mouse
6-week-old male C57BL/6 mice were subcutaneous injection with 10^6 4T1 cells to construct the 4T1 tumor bearing mouse model. After the diameter of tumor increased to 0.5 cm, the mice were utilized for the further experiments. 4T1 tumor bearing mice were randomly divided into four groups (n=3), and i.v. injected with free Cy5.5, Cy5.5 loaded PEG-lipo, Cy5.5 loaded CD-lipo and Cy5.5 loaded CB[7]-lipo at a dose of 0.5 mg/kg Cy5.5. After administration for 2 h, 4 h, 8 h ,12 h, 24 h and 48 h, the mice were anesthetized and imaged by an in vivo image system (IVIS) to evaluate the targeting delivery efficiency of these formulations. Furthermore, the heart, liver, spleen, lung, kidney, and tumor were collected after administration for 48 h, and ex vivo fluorescent signals were examined on these organs by using IVIS spectrum system for biodistribution study.

In vivo antitumor therapy of liposomes
the 4T1 tumor bearing mice were randomly divided into five group (n = 6) and i.v. administered with PBS, free DOX, blank CB-lipo, DOX loaded PEG-lipo and DOX loaded CB-lipo every three day for 4 total doses. The survival rate, body-weight change, and tumor volume were monitored every other day after first administration. At the end of the experiment, the mice were sacrificed and excised tumors were photographed and weighted. The tumor growth rate was calculated. Subsequently, the collected tumor tissues, heart tissues, lung tissues, liver tissues, spleen tissues and kidney tissues were stained with hematoxylin and eosin (HE) for histological studies. The whole blood and serum were collected for blood biochemistry and hematology analysis.

Results and Discussion

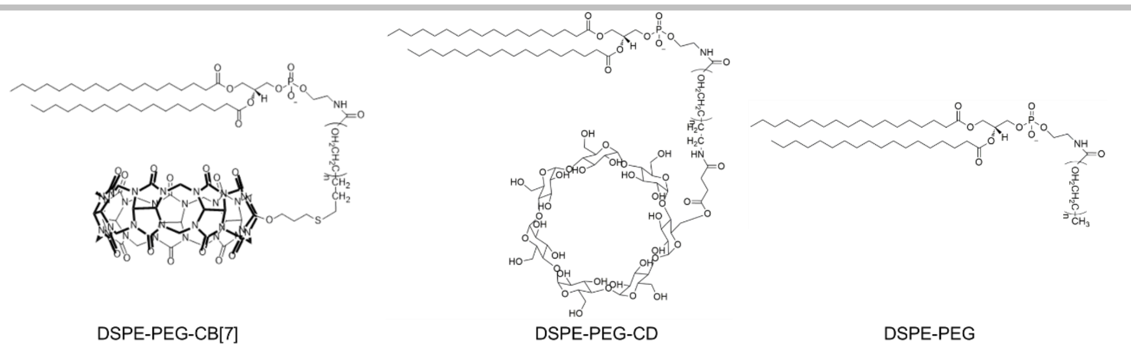

Figure S1. Chemical structure of DSPE-PEG-CB[7], DSPE-PEG-CD and DSPE-PEG.

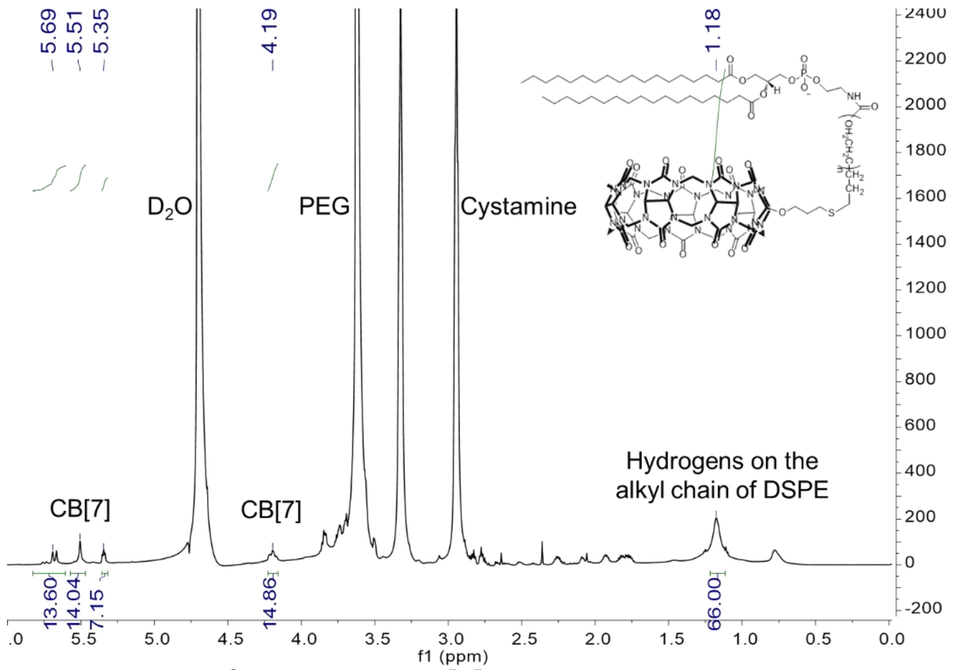

Figure S2. ^{1}H NMR spectrum of DSPE-PEG-CB[7].

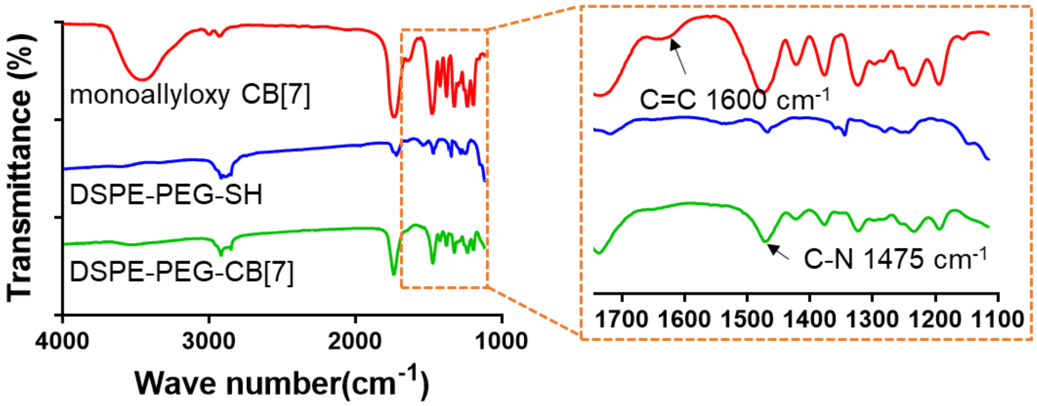

Figure S3. FT-IR spectra of DSPE-PEG-SH, monoallyloxy CB[7] and DSPE-PEG-CB[7].

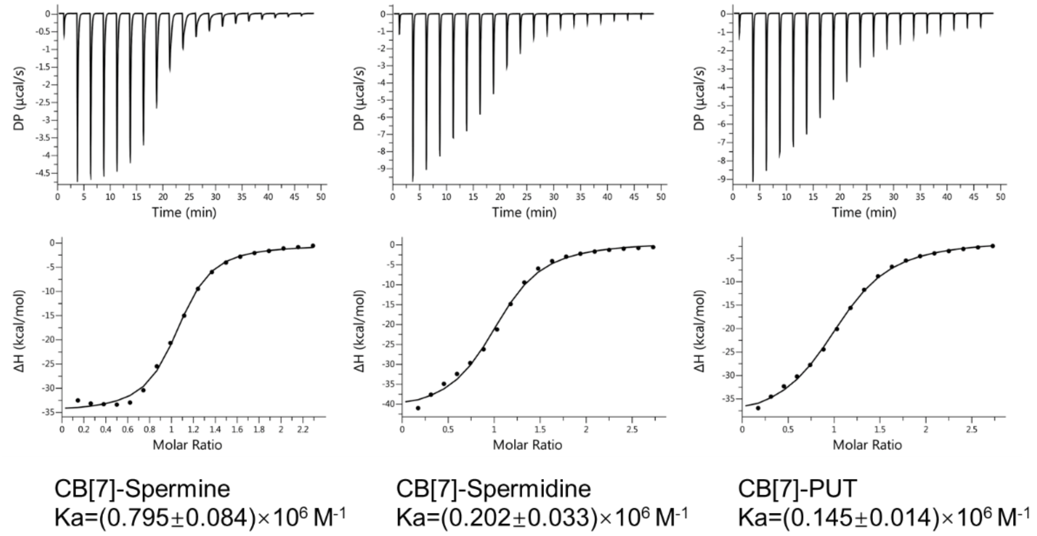

Figure S4. ITC titration results of CB[7] and SPM, SPD and PUT, respectively.

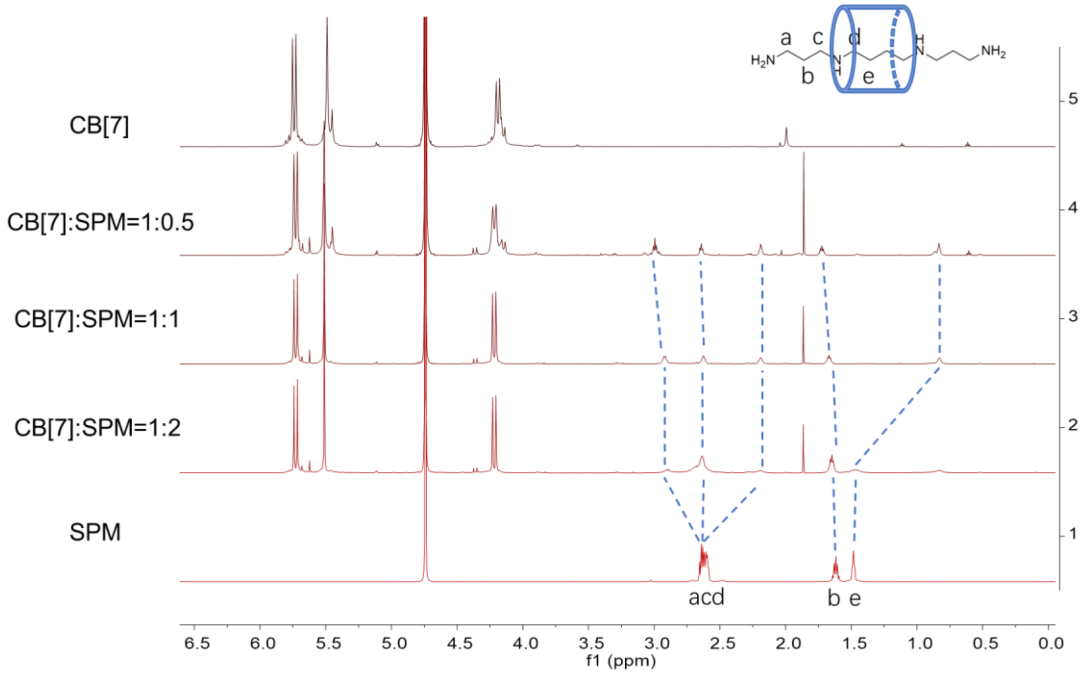

Figure S5. ^{1}H NMR spectra of CB[7], CB[7]:SPM=1:0.5, CB[7]:SPM=1:1, CB[7]:SPM=1:2 and SPM in D_2O.

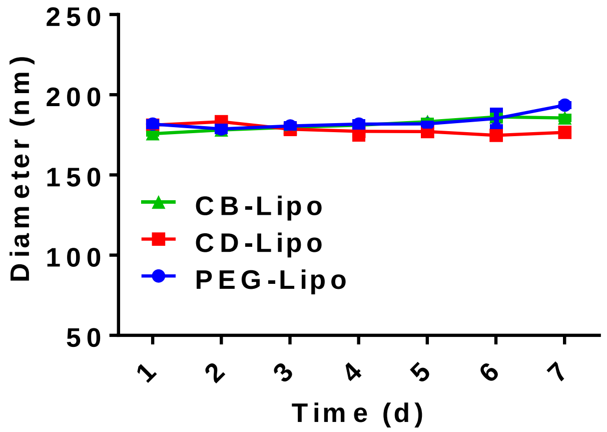

Figure S6. The size variation of PEG-lipo, CD-lipo and CB[7]-lipo, respectively, in PBS medium over 1 week.

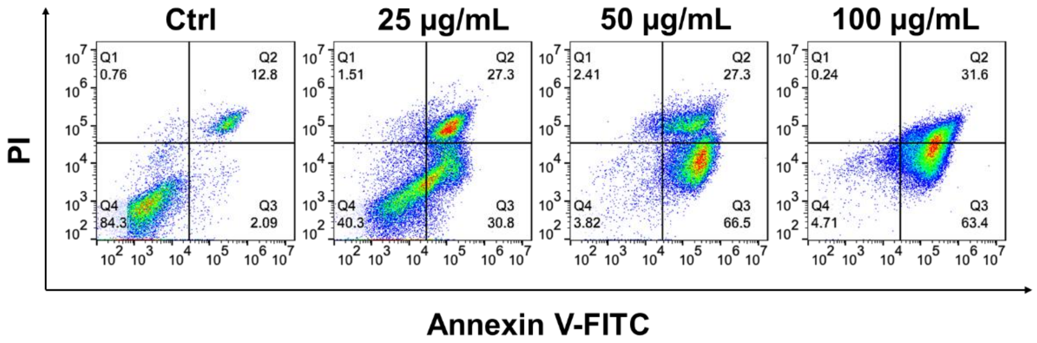

Figure S7. Apoptosis rate of 4T1 cells after incubation with CB[7]-lipo with concentration of 0, 25μg/mL, 50μg/mL, 100μg/mL, respectively, for 48 h.

IC_{50} (μg/mL)	L02	4T1
DOX	0.8441	0.8166
PEG^{DOX}-lipo	0.7976	0.7103
CD^{DOX}-lipo	0.8137	0.7920
$CB[7]^{DOX}$-lipo	0.8122	0.5047

Figure S8. IC50 values of DOX, PEG-lipo, CD-lipo and CB[7]-lipo treated L02 and 4T1 cells after 48 h.

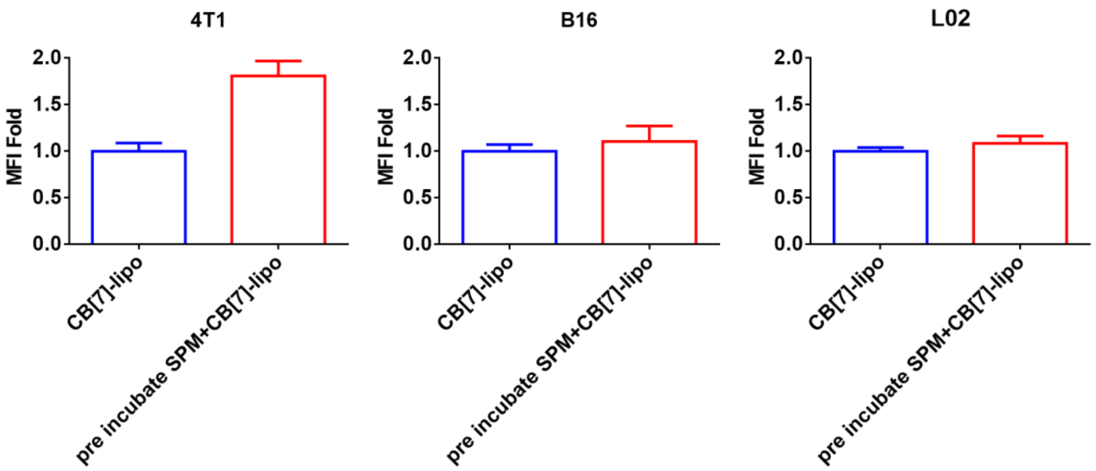

Figure S9. Mean fluorescence intensities measured by flow cytometry of 4T1 cells, B16 cells and L02 cells after incubation with Cy5.5 labeled CB[7]-lipo and pre incubate SPM for 2 h and sequentially with Cy5.5 labeled CB[7]-lipo for 2 h.

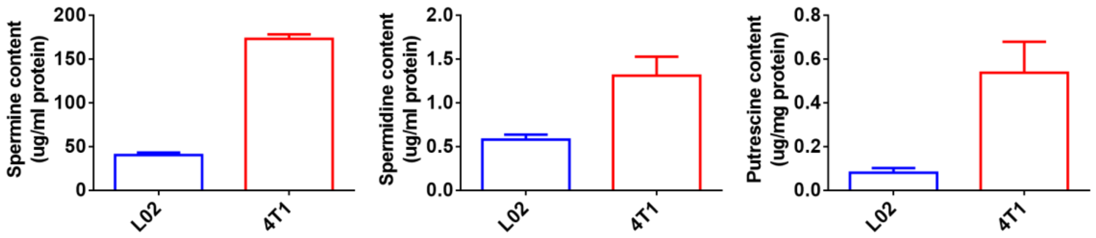

Figure S10. Intracellular SPM/SPD/PUT content in L02 cells and 4T1 cells.

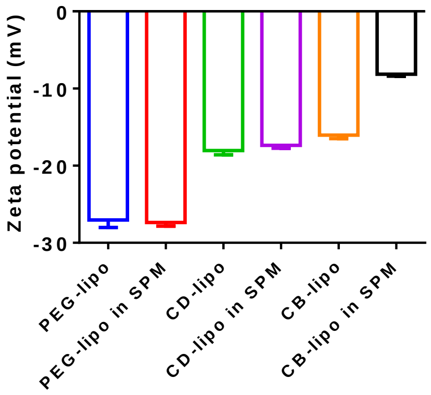

Figure S11. The zeta potential of PEG-lipo, CD-lipo and CB[7]-lipo mixed with and without SPM.

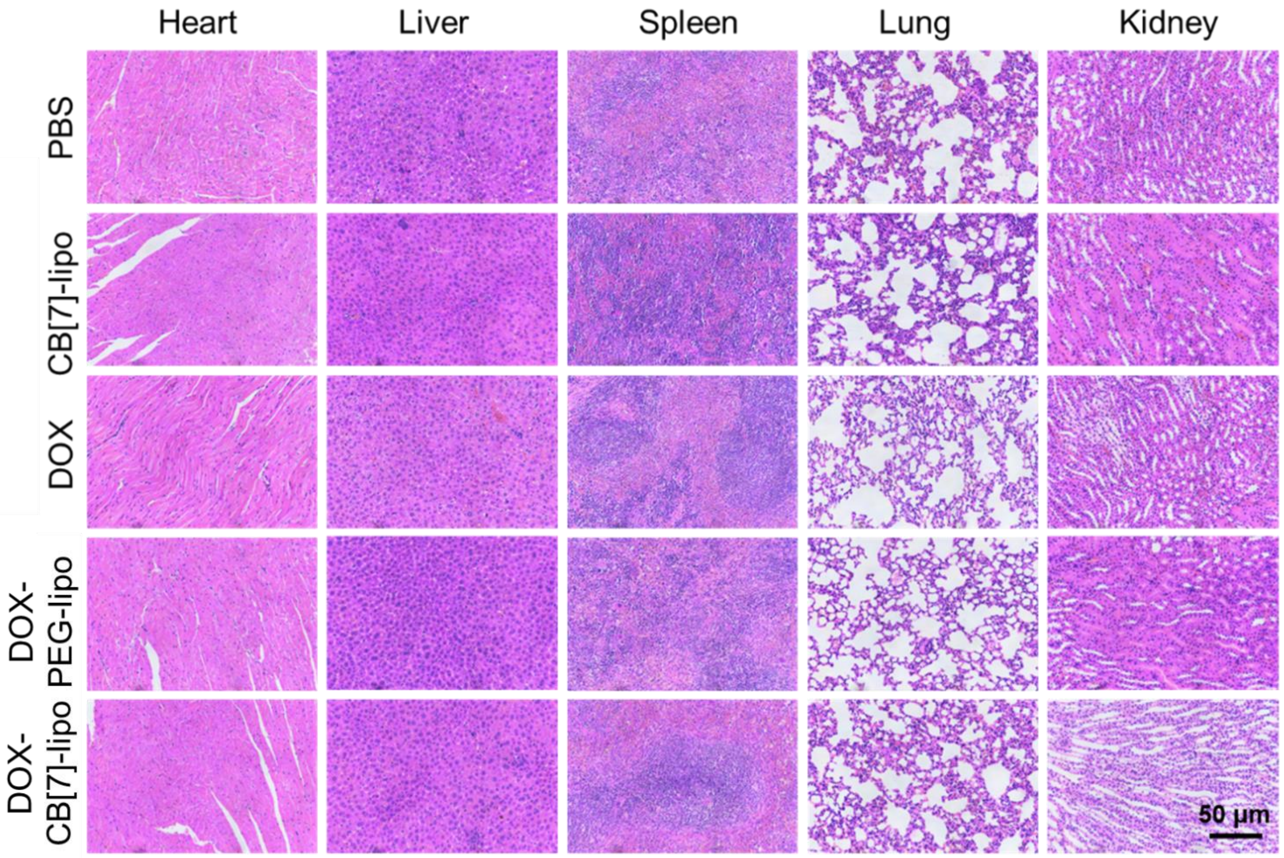

Figure S12. H&E staining images of the heart, liver, spleen, lungs and kidneys in 4T1 tumor-bearing mice sacrificed after 14-day observation.

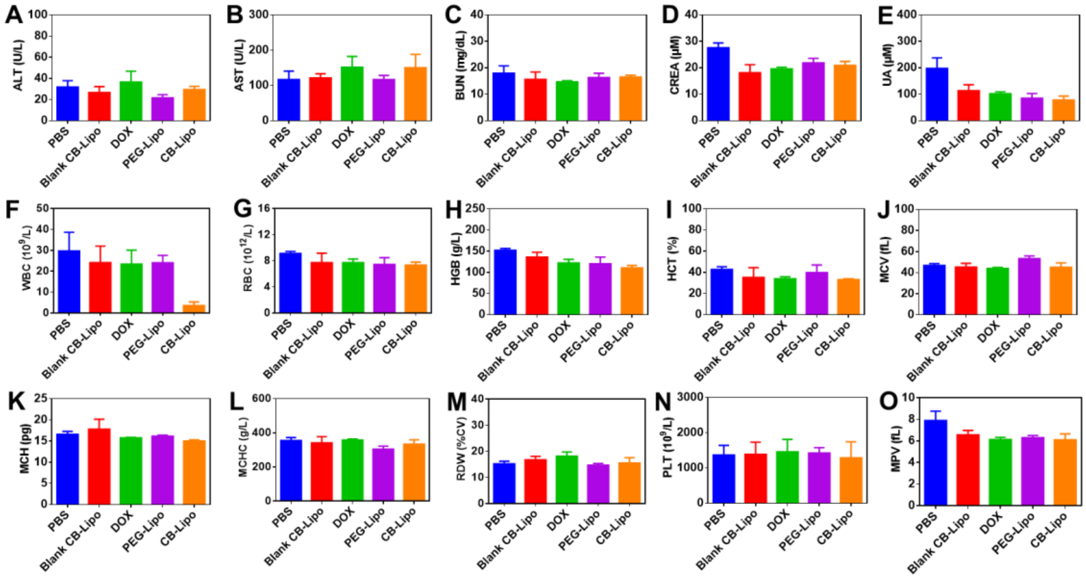

Figure S13. Blood Biochemistry and Hematology Analysis. (A-B) Quantitative analysis of liver function biomarkers (ALT and AST) and (C–E) kidney function biomarkers (BUN, CREA and UA) in the blood of the mice intravenously administrated with PBS, blank CB-lipo, DOX, PEG-lipo and CB-lipo, respectively. (F–O) The levels of (F) white blood cells, (G) red blood cells, (H) hemoglobin, (I) hematocrit, (J) mean corpuscular volume, (K) mean corpuscular hemoglobin, (L) mean corpuscular hemoglobin concentration, (M) red blood cell distribution width-standard deviation (N) platelets and (O) mean platelet volume in mice treated with PBS, blank CB[7]-lipo, DOX, PEG-lipo and CB[7]-lipo, respectively (n = 3).

References

[1] C. Gao, Q. Cheng, J. Li, J. Chen, Q. Wang, J. Wei, Q. Huang, S. M. Y. Lee, D. Gu, R. Wang, *Adv. Funct. Mater*. **2021**, *31*, 2102440.

[2] N. Penoy, B. Grignard, B. Evrard, G. Piel, *Int. J. Pharm.* **2021**, *592*, 120093.
